# Prevalence of Sinus Tract in the Patients Visiting Department of Endodontics, Kermanshah School of Dentistry

**DOI:** 10.5539/gjhs.v7n6p271

**Published:** 2015-04-23

**Authors:** Shima Sadat Miri, Omid Atashbar, Fardin Atashbar

**Affiliations:** 1 Department of Endodontics, School of Dentistry, Kermanshah University of Medical Sciences, Kermanshah, Iran; 2 School of Dentistry, Kermanshah University of Medical Sciences, Kermanshah, Iran

**Keywords:** sinus tract, prevalence, odontogenic

## Abstract

**Introduction:**

Sinus tract is one of the manifestations of chronic dental infections, which is a path for the drainage of the infection and pus. The present study was aimed to investigate the prevalence of sinus tract with dental origin analyze the correlation between sinus tract and related factors.

**Methods:**

This study was conducted on 1527 patients, visiting Kermanshah school of dentistry, in 2014.The related teeth were examined in terms of vitality test and exact location of sinus tract. Moreover, the causes of this lesion and the needs for root canal treatment were assessed in these teeth. Having obtained the data from the patients, analyzed by Mann-Whitney, Chi-square tests.

**Results:**

The frequency of sinus tract was 9.89% patients. There was a significant correlation between the prevalence of sinus tract and factors such as age, general health status, location of sinus tract and history of root canal treatment. The prevalence of sinus tract in maxilla was higher than the mandible (p=0.087). The prevalence of sinus tract in the posterior teeth (69.54%) was significantly higher than that of anterior teeth (30.46%) (p=0.000). From 724 teeth with periapical inflammation and radiolucency, 9.89% teeth had odontogenic sinus tract, and 23.42% teeth with history of root canal treatment had sinus tract.

**Conclusions:**

The most common cause of sinus tract incidence was previous root canal treatment. Therefore, clinicians need to pay a more attention to examining the posterior teeth referred for endodontic treatment.

## 1. Introduction

Sinus tract is a manifestation of chronic dental infections which provides a path for the drainage of infections and pus (Cohenca et al., 2003; Gupta & Hasselgren, 2003). The location of intra-oral or extra-oral sinus tract depends on the resistance rate against exudate extraction, distance of root apex to the outer cortical bone and bone morphology. Thus, the large distance between orifice and location of lesion is not a rare phenomenon (Gülec et al., 2001; Yasui et al., 2005; Soares et al., 2007; Magliocca et al., 2010). Intraoral sinus tract is usually found in the buccal or vestibular attached gingiva, and is indicative of the presence of chronic apical abscess and sometimes periodontal abscess (Cioffi et al., 1986). Extraoral odontogenic sinus tract can be found anywhere on the face or neck although it is mostly found on the cheek, chin, mandibular angle and sometimes floor of the nose (Spear et al., 1983). When the exodation reach the skin surface, dermal sinus tract may be mistaken for a wide range of diseases such as topical dermal infection, osteomyelitis, neoplasm, tuberculosis, actinomycosis and congenital midline sinus of upper lip (Katou & Motegi, 2009; Slutzky-Goldberg et al., 2009).

The patients with cutaneous sinus tract on the head and neck refer to the physician when they notice incorrect diagnosis and undergo unnecessary and ineffective antibiotic treatments. Unnecessary antibiotics temporarily recovered the sinus tract and the sinus tract will intensify after the antibiotic treatment is ended because the source of infection is still remained (Gupta & Hasselgren, 2003). However, the definitive treatment for drainage of sinus tract is elimination of the infection source through root canal treatment (Soares et al., 2007; Gupta et al., 2011). It has been estimated that half of the patients with oral and facial fistula undergo various dermal surgeries due to lack of proper diagnosis (Cantore et al., 2002). Early diagnosis and timely treatment of these lesions may help prevent unnecessary treatments or aggressive surgeries. Thus, it is necessary to analyze the prevalence of odontogenic sinus tract in the society in order to accelerate the clinician’s diagnosis and treatment (Gupta & Hasselgren, 2003). In addition, it is essential to obtain the necessary data concerning the epidemiology of sinus tract to plan the future treatments and to provide the opportunity to compare various populations (Sadeghi & Dibaei, 2011; Soğur et al., 2013). Furthermore, few studies have been carried out to investigate the incidence and prevalence of sinus tract. Hence, the primary objective of this study was to determine the incidence of sinus tract with dental origin in patients visiting department of endodontics at Kermanshah school of dentistry. The second objective, however, was to analyze the correlation between sinus tract and factors such as age, gender, presence of sinus tract, location of sinus tract, tooth number and history of root canal treatment.

## 2. Methods

In the present study, a total of 1527 patients (597 males and 930 females), visiting Kermanshah school of dentistry to get advice for root canal treatment over a period of 6 months, were examined by a root canal specialist. To collect the required data, a checklist (Table 1) was used which included information about age, gender, level of education, systemic diseases, presence of sinus tract, location of sinus tract, tooth number and cause of lesion (Table 1). Clinical examination included symptoms of tenderness or pain on palpation and percussion, periodontal probe and dental mobility. The teeth with root canal problem were examined in terms of the presence or lack of sinus tract. All sinus tracts were evaluated by gutta-percha and their relationship with the teeth was evaluated through radiography. As tracing is a necessary part of diagnosis without considering the research process, there is no ethical concern. The teeth were examined with regard to vital pulp test and precise location of intraoral and extraoral sinus tracts. In the case of being intraoral sinus tract, they were classified according to the position of jaw (maxilla or mandible, buccal or lingual, mesial or distal, anterior or posterior, and attached gingiva or free gingiva). The data obtained from the patients were analyzed by SPSS-18 software using Mann-Whitney, Chi-square and Fischer’s exact tests. P<0.05 was considered statistically significant.

## 3. Results

**Figure 1:**
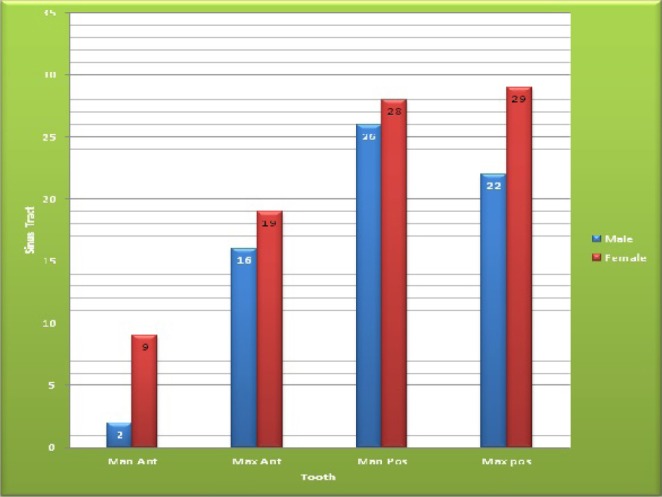
N^.^ Of sinus tracts in different teeth according to gende

From a total of 1527 patients visiting Kermanshah school of dentistry, 597 patients (39%) were male and 930 patients (61%) were female. The frequency of sinus tract was 151 patients (9.89%) out of 1527 patients (Figure 1). The prevalence of sinus tract in men was 11.05% (n=66) which was more than women with the frequency of 9.14% (n=85). There was no statistically significant difference between sinus tract and both genders. The minimum and maximum ages of the participants were 7 and 69 year old, respectively. Further, a significant correlation was found between the age group and incidence of sinus tract (p=0.029), so that the patients within the age range of 40-49 year old constituted the highest frequency. In the present study, 20.97% of sinus tracts were reported in people with elementary education, 13.33% in people with middle school education, 9.85% in people with high school education, 10.26% in people with diploma and 7.21% in people with university education. Also, there was a significant correlation between general health of the patients and incidence of sinus tract (p=0.009).The prevalence of sinus tract in maxilla was 86 people (57%), which was higher than the mandible with the prevalence of 65 patients (43%) (p=0.087) (Table 1). The prevalence of sinus tract, however, in the posterior teeth (69.54%) was significantly higher than that of anterior teeth (30.46%) (p=0.000). Sinus tract was located in buccal/labial area in all of the teeth (98.6%) except for 2 teeth (1.4%) (p=0.000) (Table 2).

**Table 2:** Distribution of the location of the sinus tracts (N=151)

	Buccal (%)		Palatinal (%)		Total	
	Count	Row N %	Count	Row N %	Count	Row N %
**Mesial of the related root**	29	100.0%	0	.0%	29	100.0%
**Midlle of the related root**	96	96.0%	4	4.0%	100	100.0%
**Distal**	18	100.0%	0	.0%	18	100.0%
**Total**	143	97.3%	4	2.7%	147	100.0%

The findings showed a statistically significant difference between free gingiva (3.12%) and attached gingiva (7.86%) in terms of distribution of sinus tract (p=0.000)(Table 3). Furthermore, the results showed that in the patients with sinus tract, distribution in 29 patients (19.5%) was mesial, in 102 patients (68.5%) medial, in 18 patients (12.1%) distal, in 132 patients (93%) apical and in 10 patients (7%) was cervical, indicating a statistically significant difference between them (p=0.000)(Table 3). In addition, 724 studied teeth (47.8%) had apical lesion from which 21% were afflicted with sinus tract and 52.2% had no sign of apical lesion. Also, 63 out of 269 teeth with history of root canal treatment had sinus tract (p=0.000). The findings also indicated a strong correlation between sinus tract and was previous root canal treatment. From 269 cases with previous endodontic treatment, 63 cases (23.4%) and from 1255 cases without previous endodontic treatment, 88 cases (7.01%) had sinus tracts.

**Table 3:** Distribution of the location of the sinus tracts (N=151)

	Attachment Gingivall		Free gingival		Total	
		
	Count	Row N %	Count	Row N %	Count	Row N %
**Mesial of the related root**	28	96.6%	1	3.4%	29	100.0%
**Midlle of the related root**	90	90.9%	9	9.1%	99	100.0%
**Distal of the related root**	10	55.6%	8	44.4%	18	100.0%
**Total**	128	87.7%	18	12.3%	146	100.0%

## 4. Discussion

This study was carried out from early march to the late October to investigate the prevalence of sinus tract among the patients referring to the department of endodontics at Kermanshah school of dentistry and to analyze a number of predisposing factors associated with it.

The incidence rate of this lesion was 9.89% in the present study which is partly in line with the figures reported in several studies. Also, there was no statistically significant correlation between incidence of sinus tract and gender, which is in line with the results of Soğur (Soğur et al., 2013), Sadeghi (Sadeghi & Dibaei, 2011) and Goldberg (Slutzky-Goldberg et al., 2009). There was a significant correlation between the age group and prevalence of sinus tract (p<0.05), so that the risk of sinus tract in 40-49 year-old patients was approximately 0.44 times greater than the patients under 20 years old (p=0.029). The study group with highest percent of sinus tract were in the 40-49 year age group. There are few reports about the prevalence pattern of oral sinus tract in which no agreement has been shown on the most commonsite of sinus tract involvement. In this study the frequency of sinus tract between maxilla and mandible was not statistically significant (p=0.087), but the incidence of sinus tract in the molar area (51% of all cases) was significantly higher than other areas, and in the lower molars was twice greater than the upper molars. In the present study, none of the sinus tracts was related to mandibular canines. According to the study carried out by Goldberg (Slutzky-Goldberg et al.,2009) on 1119 patients aged 7-75, the most common involved area was reported to be mandibular premolar(Slutzky-Goldberg et al., 2009); however, no incidence of sins tract was reported in maxillary and mandibular canine. In a study performed by Soğur (Soğur et al., 2013) on 499 patients aged 15-70, a higher involvement of molars was reported. It should be noted that in the study of Soğur (Soğur et al., 2013) no incidence of sinus tract was reported in the mandibular canine. First molars are the first permanent teeth that grow in the mouth and are more vulnerable to carries and consequently root canal treatment or extraction. Thus, high incidence of sinus tract is expected to occur in the posterior teeth (Soğur et al., 2013). In contrast with the findings of the present study, Sadeghi (Sadeghi & Dibaei, 2011) reported the highest rate of incidence for this lesion in the mandibular anterior teeth in the age range of 10-19. The high incidence of dental trauma in the anterior teeth of the young people may be associated with the prevalence of sinus tract in the younger ages (Sadeghi & Dibaei, 2011). The findings of the present study indicated a statistically significant difference between intraoral and extraoral sinus tract distribution (p=0.000), and 98.7% of sinus tracts were intraoral. Moreover, the analysis of the prevalence of buccal/lingual sinus tract showed that the frequency of sinus tract was higher in the buccal area than in lingual area, which is in line with the findings of Gupta (Gupta & Hasselgren, 2003) and Soğur (Soğur et al., 2013). This can be due to the fact that the alveolar bone in lingual/palatal area is generally more compact than in the buccal/labial bone (Valderhaug, 1973). Furthermore, the most common site of sinus tract was observed in the medial area of the teeth in the patients with sinus tract. Among the existing studies, only the study carried out by Mert reported distal area as the most common area for sinus tract. This difference, however, can be due to anatomic variations. In the present study, 132 patients (93%) had sinus tract in the apical area, which is in agreement with the results of Goldberg. Moreover, 224 participants were afflicted with systemic disease, 4.9% of whom had sinus tract. This is in contrast with the findings of the studies by Goldberg (Slutzky-Goldberg et al., 2009) and Soğur et al. (2013) in which no significant correlation was reported between the incidence of sinus tract and systemic diseases. Also, 47.8% of the studied teeth had apical trauma in the present study, from which 21% suffered from sinus tract and 63 patients (4.23%) had sinus tract from 269 patients with history of root canal treatment. Since the patients with odontogenic sinus tract undergo unnecessary and inappropriate treatments due to incorrect diagnosis, early diagnosis and appropriate root canal treatment provide a permanent solution to this problem. Hence, it is necessary for the clinicians to be aware of the prevalence and alternating locations of odontogenic sinus tract for the sake of rapid diagnosis and appropriate treatment which result in quick amelioration of thislesion.

## 5. Conclusion

It is necessary for the clinicians to be aware of the prevalence and alternating locations of odontogenic sinus tract for the sake of rapid diagnosis and appropriate treatment which result in quick amelioration of this lesion.
